# CLEAR Lenticule Extraction for Enhancement After Primary Lenticule Extraction Surgery

**DOI:** 10.3390/jcm15135036

**Published:** 2026-06-28

**Authors:** Sungho Choi, Yoonseong Choi, Deok Jo Nam

**Affiliations:** 1 First Samsung Eye Clinic, Seoul 06621, Republic of Korea; londjve@naver.com; 2 College of Arts and Science, New York University, New York, NY 10012, USA; yoonseongcys@gmail.com

**Keywords:** CLEAR, LDV Z8, KLEx, lenticule extraction, retreatment

## Abstract

**Background**: We aimed to evaluate the feasibility, safety, and efficiency of Corneal Lenticule Extraction for Advanced Refractive Correction (CLEAR) enhancement performed after primary CLEAR. **Methods**: Six eyes from five patients underwent enhancement to correct residual myopic error. All procedures were carried out with the low-energy FEMTO LDV Z8 laser platform (Ziemer Ophthalmic Systems AG, Port, Switzerland) equipped with integrated optical coherence tomography enabling precise lenticule positioning. **Results**: The myopic residual refractive sphere was fully corrected in all cases, with refraction remaining stable over the follow-up period. All eyes showed improvement in uncorrected distance visual acuity after retreatment with CLEAR and achieved 20/20 vision or better at the final follow-up visit. **Conclusions**: Our results suggest that CLEAR enhancement is a safe and effective option for treating residual myopic refractive error after primary CLEAR, keeping the procedure flapless.

## 1. Introduction

Surgical enhancement after refractive surgery is indicated when residual refractive error results in suboptimal uncorrected visual acuity or patient dissatisfaction, despite otherwise successful primary treatment outcomes. Such residual errors may reduce visual quality, induce symptoms such as glare or blur, and represent one of the leading causes of secondary intervention following corneal refractive procedures [1,2,3]. Several surgical options are available for enhancement after keratorefractive lenticule extraction (KLEx), including surface ablation techniques such as photorefractive keratectomy (PRK), flap-based approaches using thin- or thick-flap laser-assisted in situ keratomileusis (LASIK), and cap-to-flap conversion procedures such as CIRCLE. Each of these techniques presents distinct advantages and limitations in terms of biomechanical impact, healing response, refractive predictability, and risk of complications such as haze formation, flap-related issues, or regression [4,5,6,7].

More recently, lenticule extraction techniques have also been explored as enhancement strategies. A case report demonstrated that correction of residual myopic refractive error after prior PRK can be successfully achieved using corneal lenticule extraction for advanced refractive correction (CLEAR) [8], a newer femtosecond laser-based lenticule extraction procedure introduced after small-incision lenticule extraction (SMILE) [9,10].

The concept of performing a secondary lenticule extraction within a previously treated cornea was first introduced by Donate and Thaëron in 2015, who reported the feasibility of performing SMILE beneath the interface of a prior SMILE procedure [11]. This approach demonstrated that repeat intrastromal lenticule creation is technically possible, provided that the new lenticule can be accurately positioned relative to the primary interface. Subsequently, Sedky et al. reported that cap-preserving SMILE enhancement can effectively reduce residual myopic refractive error following primary SMILE, particularly in highly myopic eyes [12]. A major advantage of secondary KLEx procedures as retreatment options is the ability to maintain a flapless approach, thereby avoiding surface ablation and preserving both Bowman’s layer and the integrity of the anterior stromal architecture. This may contribute to improved biomechanical stability and a more favorable wound healing response compared with flap-based or surface ablation techniques [13,14]. Importantly, these early studies consistently emphasized that precise geometric alignment between the primary and secondary lenticule interfaces is critical to achieving accurate refractive correction, as even minor decentration or depth mismatch may result in suboptimal visual outcomes or induction of higher-order aberrations [12,15]. The CLEAR procedure performed with the low-energy FEMTO LDV Z8 femtosecond laser platform (Ziemer Ophthalmic Systems AG, Port, Switzerland) incorporates a proprietary three-dimensional spectral-domain optical coherence tomography (OCT) system that enables high-resolution intraoperative imaging of corneal structures [16]. This integrated imaging capability represents a potential advantage for enhancement procedures requiring precise spatial localization within previously treated corneal tissue. Intraoperative OCT imaging during CLEAR surgery allows real-time visualization of the corneal layers and prior treatment interfaces, facilitating accurate positioning of the secondary lenticule relative to the primary interface in both depth and centration. This capability may reduce reliance on external marking systems and improve the reproducibility of enhancement procedures. To date, no published studies have specifically evaluated the use of CLEAR as an enhancement procedure following primary CLEAR surgery, and clinical data on repeat lenticule extraction using the same platform remain limited. The aim of this study was therefore to investigate the feasibility, safety, and efficacy of CLEAR enhancement following primary CLEAR, with a particular focus on refractive predictability, visual outcomes, and the role of intraoperative OCT in achieving accurate lenticule alignment.

## 2. Materials and Methods

In this case series, six eyes of five patients who underwent CLEAR between June 2022 and September 2022 and required correction of postoperative residual myopic refractive error were reviewed retrospectively. No patient had severe ocular surface diseases, keratoconus, or cataracts. The criteria for inclusion were (1) residual, correctable refractive error associated with postoperative uncorrected distance visual acuity (UDVA), causing patient dissatisfaction; (2) postoperative refractive stability; and (3) residual stromal thickness of at least 250 µm. Eyes were considered for secondary lenticule extraction only when preoperative anterior-segment OCT confirmed that the predicted residual stromal thickness—calculated from the cap thickness, residual stromal bed, and the thickness of tissue to be removed by the secondary procedure—remained within accepted safety limits, and only a small magnitude of residual correction was required. When the predicted residual stromal thickness fell below this threshold, surface ablation (LASEK/PRK) was performed instead. The CLEAR enhancement procedure was performed after 12 to 32 weeks (from October 2022 to February 2023) after the initial CLEAR treatment, all performed by a single experienced surgeon and using the low-energy FEMTO LDV Z8 femtosecond laser. All patients received a routine preoperative ophthalmic assessment, which included UDVA and corrected distance visual acuity (CDVA), manifest refraction, slit-lamp examination, keratometry, Scheimpflug-based corneal topography (Pentacam HR, Oculus Optikgeräte GmbH, Wetzlar, Germany), and corneal OCT (Cirrus HD-OCT, Carl Zeiss Meditec, Dublin, CA, USA).

**Figure 1:**
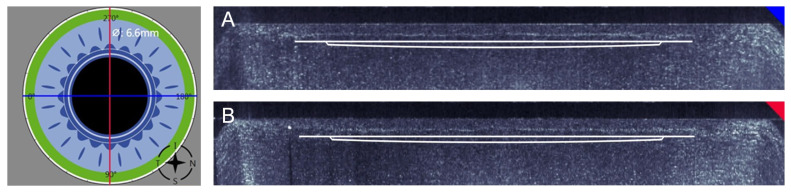
Intraoperative optical coherence tomography (OCT) images. The surgeon should verify a precise geometrical alignment between the virtual image of the lenticule enhancement procedure cut and the initial treatment interface along the horizontal meridian (**A**) and along the vertical meridian (**B**).

The requirement for informed consent of this study was waived because of the retrospective design. This study was approved by the Institutional Review Board of First Samsung Eye Clinic (No. FSEC-202404-HRR002-02) and adhered to the Tenets of the Declaration of Helsinki. A swept-source optical coherence tomography biometer (SS-OCT, Argos^®^, Alcon Laboratories, Inc., Fort Worth, TX, USA) was used to obtain a reference image, which was transferred to a digital marker (Verion^®^, Alcon Laboratories, Inc., Fort Worth, TX, USA) for verification of the astigmatism axis. For secondary CLEAR, the programmed cap thickness was 30 μm thicker than for primary CLEAR treatment. The programmed optical zone diameter was set to 6.5 mm (same as for primary CLEAR), and a 2.0 mm lenticule access incision was created 30° away from the initial treatment incision. Before docking the laser handpiece, four corneal reference marks were placed at the horizontal and vertical meridians, 7.5 mm apart, using an image-guided system (Verion^®^, Alcon) under the surgical microscope. These marks were aligned to bisect the first Purkinje image. Intraoperative anterior segment imaging was performed with the built-in OCT, and anatomical landmarks were used to position the new lenticule under the primary treatment interface (Figure 1). The lenticule extraction surgical procedure was performed using the same standard technique as in the primary CLEAR treatment [17]. Although the smaller corrections treated with secondary CLEAR produced thinner lenticules, dissection was complete and uneventful in all cases. Postoperative examinations were repeated at 1 week, 1 month, and 3 months postoperatively. The last reported follow-up visit occurred between 6 and 14 months postoperatively. No intraoperative or postoperative complications were observed during or after primary CLEAR and CLEAR enhancement procedures. Detailed parameters of the primary CLEAR treatment are provided in Table S1, and the tomographic measurements obtained before the primary CLEAR, before the secondary CLEAR, and after the secondary CLEAR are provided in Table S2 (Supplementary Materials).

## 3. Statistical Analysis

Given the small sample size of this case series (six eyes of five patients), the data were assumed not to be normally distributed and were insufficient for robust inferential statistical analysis. Accordingly, continuous variables were summarized using descriptive statistics as the mean ± standard deviation and range, and no formal inferential or normality testing was performed; pre- and postoperative outcomes are reported descriptively.

## 4. Results

**Figure 2:**
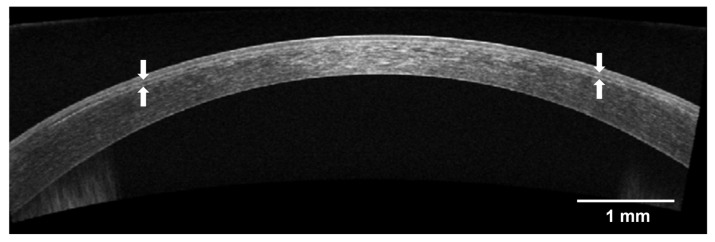
OCT image after a CLEAR enhancement procedure. Two lenticule interfaces (arrows) can be observed on the OCT image.

Preoperative characteristics prior to the primary CLEAR treatment are summarized in Table 1. Before enhancement (Table 2), the manifest refraction spherical equivalent (MRSE) ranged from −0.75 to −1.25 D, with a mean of −1.04 ± 0.18 D. All eyes demonstrated residual low myopic refractive error associated with reduced uncorrected distance visual acuity (UDVA), with pre-enhancement UDVA ranging from 0.4 to 1.0 (decimal scale), while corrected distance visual acuity (CDVA) remained 1.2–1.5 in all cases. The parameters of the secondary CLEAR enhancement procedure, including the interval between the primary and secondary treatments and the programmed cap thickness, are summarized in Table 3. Following enhancement, refractive outcomes showed a consistent shift toward emmetropia across all eyes (Table 4). At postoperative month 3, MRSE ranged from plano to +0.75 D, with a mean of +0.32 ± 0.28 D. At final follow-up (6–14 months), MRSE ranged from −0.25 to +0.38 D, with most eyes clustered within ±0.25 D of emmetropia. At final follow-up, 5/6 eyes (83%) were within ±0.50 D and 6/6 eyes (100%) were within ±1.00 D of emmetropia. Refractive stability was maintained between month 3 and the last follow-up visit, with no clinically relevant regression observed in any case. UDVA improved in all eyes following enhancement. At month 3, UDVA ranged from 0.9 to 1.5 (decimal), with 4/6 eyes achieving 1.0 or better. At final follow-up, UDVA ranged from 1.0 to 1.5, with all eyes achieving 20/20 equivalent or better. CDVA remained stable or improved in all cases, ranging from 1.0 to 1.5 at final follow-up, with no eye losing lines of CDVA. One eye (Patient 4) demonstrated transient postoperative corneal edema with associated mild visual reduction at week 1, which resolved spontaneously and did not affect final visual or refractive outcomes. Inter-eye variability remained low despite differences in baseline refractive error (−4.25 to −7.63 D pre-primary surgery; Table 1) and corneal parameters. Outcomes were consistent across the range of treated myopic errors and residual stromal thickness values (264–291 µm at enhancement; Table 3). No intraoperative complications occurred. In all cases, the primary lenticule interface was successfully identified and secondary lenticule dissection was completed without technical difficulties. No cases of cap-related complications, epithelial ingrowth, infection, or interface irregularities were observed. Postoperative anterior segment OCT confirmed the presence of two distinct lenticule interfaces without signs of abnormal corneal remodeling (Figure 2).

## 5. Discussion

The reported incidence of retreatments after refractive lenticule extraction ranges from 2.1% to 4%, indicating that although primary procedures are generally predictable, a clinically relevant subset of patients still requires secondary intervention to achieve optimal refractive outcomes [1,18]. This highlights the importance of establishing safe and effective enhancement strategies specifically tailored to lenticule-based procedures. In a study of 524 eyes treated with SMILE, most enhancements (78.6%) were performed due to primary undercorrection, suggesting that residual myopia remains the predominant indication for retreatment following lenticule extraction procedures [1]. This may reflect limitations in nomogram accuracy, individual biomechanical variability, or intraoperative factors affecting lenticule extraction. Similarly, in the present case series, all treated eyes underwent enhancement for residual myopic undercorrection, reinforcing the observation that this remains the most common indication for secondary intervention after primary CLEAR. Several risk factors have been associated with the need for enhancement surgery after lenticule extraction, including older patient age, higher degrees of preoperative myopia or astigmatism, and intraoperative events such as suction loss [1]. These factors may influence both the accuracy of the initial refractive correction and the corneal healing response, thereby increasing the likelihood of residual refractive error. Several surgical strategies have been proposed for enhancement after KLEx, including LASIK-based approaches in which a secondary flap is created either anterior or posterior to the original cap, depending on its thickness and the epithelial profile [10,19,20]. While these approaches allow effective refractive correction, they introduce additional complexity in surgical planning and carry inherent flap-related risks. The use of LASIK as a retreatment modality is associated with specific intraoperative risks, including flap-related complications such as buttonhole formation and gas bubble breakthrough into the flap interface, which may compromise surgical outcomes and increase the risk of visual disturbances [21]. Surface ablation techniques, particularly PRK, represent another commonly used option for enhancement after KLEx, especially in cases where flap creation is undesirable or contraindicated [1,19]. PRK enhancement has been shown to be both safe and effective following primary lenticule extraction, with the additional advantage of preserving corneal biomechanical integrity by avoiding further stromal dissection [1,13,22]. This makes it an attractive option in cases where residual stromal thickness is limited. However, PRK is associated with several limitations, including the risk of refractive regression, increased postoperative inflammation, delayed visual recovery, and the potential for late corneal haze formation, particularly in higher corrections [23,24]. Accordingly, repeat lenticule extraction may be particularly well suited to patients who prioritize rapid visual recovery and who retain sufficient residual stromal thickness to safely accommodate the thicker secondary cap, whereas patients with limited residual stroma remain better candidates for surface ablation. Surface ablation such as transepithelial PRK has been reported as a safe and effective option for enhancement after primary lenticule extraction [5,22]. In selected patients, however, a repeat lenticule-based approach offers distinct advantages. The integrated intraoperative OCT of the FEMTO LDV Z8 enables creation of the secondary lenticule at a precisely defined depth and position relative to the prior interface, and in our experience a separation of approximately 30 µm allows smooth dissection and safe lenticule removal while preserving the residual stromal bed. Because all enhancements involved small corrections and were performed only when the residual stroma remained within safe limits, biomechanical risk was minimized. A flapless lenticule enhancement may also be preferred by patients seeking more rapid visual recovery than surface ablation provides. Secondary CLEAR is therefore proposed not as a universal alternative, but as one OCT-guided option selected after careful biomechanical assessment. In addition to PRK and LASIK, alternative approaches such as cap-to-flap conversion (e.g., CIRCLE) and secondary lenticule extraction have been proposed as retreatment strategies, each offering distinct advantages in terms of surgical approach and tissue preservation. Experimental studies comparing CIRCLE, secondary SMILE, and surface ablation have demonstrated that each retreatment modality induces distinct corneal wound healing and tissue response patterns, suggesting that the choice of enhancement technique may influence long-term corneal stability and optical quality [14]. A secondary KLEx procedure, by maintaining a flapless approach, may offer theoretical advantages over alternative techniques, including improved preservation of corneal structural integrity, reduced wound healing response, and better maintenance of keratocyte density within the anterior stroma [14]. These factors may contribute to more stable and predictable refractive outcomes. One technical limitation of secondary lenticule extraction is that smaller refractive corrections result in thinner lenticules, which can be more difficult to dissect and extract, potentially increasing surgical complexity and the risk of incomplete tissue removal. Previous studies on SMILE enhancement have emphasized that precise positioning of the secondary lenticule relative to the primary interface is a critical determinant of surgical success, as even minor misalignment can lead to residual refractive error or induction of higher-order aberrations [12]. To address this challenge, specialized tools such as the Sedky SMILE Retreatment Centering Marker have been developed to assist in achieving accurate centration of the secondary lenticule relative to the primary interface, highlighting the technical difficulty of alignment in repeat lenticule procedures [12]. In the present study, the secondary lenticule was deliberately created posterior to the primary treatment interface, allowing spatial separation between the two planes and minimizing the risk of interface interaction. This posterior positioning strategy avoided mechanical interference between the primary and secondary interfaces while preserving sufficient residual stromal thickness, which is a critical factor for maintaining corneal biomechanical stability and minimizing the risk of ectasia [25]. Because the enhancement procedure involves the creation of a thicker cap compared with the primary treatment, careful preoperative evaluation of residual stromal thickness is essential to ensure that safe biomechanical limits are maintained. In this study, precise geometric alignment between the primary and secondary lenticule interfaces was facilitated by the integrated three-dimensional spectral-domain OCT system of the FEMTO LDV Z8 platform, enabling direct visualization of corneal structures during surgical planning. The intraoperative visualization of a virtual lenticule overlay provided by the CLEAR software allowed the surgeon to accurately position the secondary treatment relative to anatomical landmarks of the primary interface, improving both centration and depth alignment and reducing reliance on external marking systems. Furthermore, the stable suction characteristics of the CLEAR platform reduce the risk of intraoperative suction loss, allowing sufficient time for meticulous OCT-guided positioning and thereby potentially improving surgical precision and reproducibility. The clinical outcomes observed in this series were favorable, with all treated eyes achieving uncorrected and corrected distance visual acuity of at least 20/20 at the final follow-up, indicating effective correction of residual refractive error and good visual recovery. It is possible that outcomes following CLEAR enhancement may be influenced by patient age, as younger individuals tend to exhibit more predictable corneal healing responses and refractive stability, potentially leading to improved efficacy compared with older populations [26]. This study is limited by the absence of a direct comparison with alternative enhancement techniques such as PRK or LASIK, which precludes definitive conclusions regarding the relative advantages of CLEAR enhancement over established methods. Additionally, the small sample size limits the ability to perform meaningful statistical analyses or to generalize the findings to a broader patient population. Finally, the number of reported outcome measures is limited, with no detailed analysis of higher-order aberrations, contrast sensitivity, or patient-reported visual quality. In conclusion, this study provides initial proof-of-concept that CLEAR enhancement following primary CLEAR is both feasible and clinically effective, supporting its potential role as a viable retreatment option for residual refractive error while maintaining a flapless surgical approach [11,12]. The integration of intraoperative OCT imaging and stable suction within the FEMTO LDV Z8 platform appears to play a key role in enabling precise positioning of the secondary lenticule, which is likely a critical factor in achieving accurate and reproducible refractive outcomes.

## Figures and Tables

**Table 1:** Preoperative Characteristics.

Patient	Eye Side	UDVA	CDVA	Sphere	Cylinder	MRSE
		(Decimal)	(Decimal)	(D)	(D)	(D)
Patient 1 (M, 34 years)	OD	0.05	1.5	−4.00	−0.50	−4.25
Patient 2 (F, 29 years)	OD	0.05	1.5	−4.75	−0.50	−5.00
Patient 3 (M, 30 years)	OD	0.05	1.5	−5.00	−0.50	−5.25
	OS	0.05	1.5	−5.00	−0.25	−5.13
Patient 4 (M, 24 years)	OD	0.01	1.2	−6.25	−0.50	−6.50
Patient 5 (M, 20 years)	OD	0.03	1.2	−6.75	−1.75	−7.63

UDVA = uncorrected distance visual acuity; CDVA = corrected distance visual acuity; MRSE = manifest refraction spherical equivalent.

**Table 2:** Refractive and visual outcomes prior to the secondary CLEAR treatment.

Patient	Eye Side	UDVA	CDVA	Sphere	Cylinder	MRSE
		(Decimal)	(Decimal)	(D)	(D)	(D)
Patient 1	OD	0.7	1.2	−0.75	−0.50	−1.00
Patient 2	OD	0.8	1.2	−1.00	0.00	−1.00
Patient 3	OD	1.0	1.2	−0.50	−1.00	−1.00
	OS	0.4	1.2	−0.50	−1.50	−1.25
Patient 4	OD	0.9	1.5	−0.50	−0.50	−0.75
Patient 5	OD	0.8	1.5	−1.00	−0.50	−1.25

UDVA = uncorrected distance visual acuity; CDVA = corrected distance visual acuity; MRSE = manifest refraction spherical equivalent.

**Table 3:** Parameters of the CLEAR enhancement procedure.

Patient	Time Between Primary and Secondary CLEAR	Eye Side	Mean Sim-K (D)	Q Value	Thinnest Pachymetry (µm)	Optical Zone (mm)	Cap Thickness (µm)	Residual Stroma * (µm)
Patient 1	19 weeks	OD	39	0.31	488	6.5	150	291
Patient 2	12 weeks	OD	38.2	0.16	500	6.5	150	286
Patient 3	13 weeks	OD	36.1	0.52	489	6.5	145	282
		OS	35.4	0.41	484	6.5	145	264
Patient 4	32 weeks	OD	38.5	0.46	517	6.5	155	284
Patient 5	28 weeks	OD	36.6	0.55	469	6.5	140	271

* Residual stroma displayed on the FEMTO LDV Z8 monitor.

**Table 4:** Refractive and visual outcomes after the CLEAR enhancement procedure.

Patient	Eye Side	UDVA	CDVA	Sphere	Cylinder	MRSE
		(Decimal)	(Decimal)	(D)	(D)	(D)
		Postoperative Week 1				
Patient 1	OD	0.6	0.6	0.00	−0.50	−0.25
Patient 2	OD	N/A	N/A	N/A	N/A	N/A
Patient 3	OD	1.2	1.2	0.50	−0.50	0.25
	OS	1.2	1.2	1.00	−0.50	0.75
Patient 4	OD	0.6	0.9	1.00	−0.50	0.75
Patient 5	OD	N/A	N/A	N/A	N/A	N/A
		Postoperative Month 1				
Patient 1	OD	N/A	N/A	N/A	N/A	N/A
Patient 2	OD	1.2	1.5	1.00	0.00	1.00
Patient 3	OD	N/A	N/A	N/A	N/A	N/A
	OS	N/A	N/A	N/A	N/A	N/A
Patient 4	OD	N/A	N/A	N/A	N/A	N/A
Patient 5	OD	1.0	1.2	0.25	−0.25	0.13
		Postoperative Month 3				
Patient 1	OD	1.0	1.0	0.25	−0.50	0.00
Patient 2	OD	1.5	1.5	0.50	−0.25	0.38
Patient 3	OD	1.2	1.2	0.50	−0.50	0.25
	OS	1.2	1.2	1.00	−0.50	0.75
Patient 4	OD	0.9	1.2	1.00	−0.50	0.75
Patient 5	OD	1.2	1.2	0.50	−0.25	0.38
		Last follow-up (Postoperative Month 6–14)				
Patient 1	OD	1.0	1.0	0.00	−0.50	−0.25
Patient 2	OD	1.5	1.5	0.50	−0.25	0.38
Patient 3	OD	1.2	1.2	0.25	0.00	0.25
	OS	1.5	1.5	0.50	−0.25	0.38
Patient 4	OD	1.2	1.2	0.00	0.00	0.00
Patient 5	OD	1.2	1.2	0.00	−0.25	−0.13

UDVA = uncorrected distance visual acuity; CDVA = corrected distance visual acuity; MRSE = manifest refraction spherical equivalent.

## Data Availability

The original contributions presented in this study are included in the article/Supplementary Materials. Further inquiries can be directed to the corresponding author.

## Supplementary Materials

The following supporting information can be downloaded at https://www.mdpi.com/article/10.3390/jcm15135036/s1, Supplementary Table S1. Parameters of the primary CLEAR treatment. Supplementary Table S2. Tomography parameters measured preoperatively to primary CLEAR, preoperatively to secondary CLEAR, and postoperatively to secondary CLEAR.

## References

[1] Liu Y.C., Rosman M., Mehta J.S. (2017). Enhancement after Small-Incision Lenticule Extraction: Incidence, Risk Factors, and Outcomes. Ophthalmology.

[2] Joshi R.S., Singh H., Joshi R.R. (2025). Beyond the First Cut: Evaluating LASIK Reoperation Trends. Rom. J. Ophthalmol..

[3] Moshirfar M., Santos J.M., Wang Q., Stoakes I.M., Porter K.B., Theis J.S., Hoopes P.C. (2023). A Literature Review of the Incidence, Management, and Prognosis of Corneal Epithelial-Related Complications After Laser-Assisted In Situ Keratomileusis (LASIK), Photorefractive Keratectomy (PRK), and Small Incision Lenticule Extraction (SMILE). Cureus.

[4] Moshirfar M., Shah T.J., Masud M., Linn S.H., Ronquillo Y., Hoopes P.C. (2018). Surgical options for retreatment after small-incision lenticule extraction: Advantages and disadvantages. J. Cataract Refract. Surg..

[5] Moshirfar M., Parsons M.T., Chartrand N.A., Lau C.K., Stapley S., Bundogji N., Ronquillo Y.C., Hoopes P.C. (2022). Photorefractive Keratectomy Enhancement (PRK) After Small-Incision Lenticule Extraction (SMILE). Clin. Ophthalmol..

[6] Ye Y., Hou X., Yu N., Chen P., Zhuang J., Yu K. (2024). Corneal Epithelial Remodeling Induced by Photorefractive Keratectomy Enhancement After Small-Incision Lenticule Extraction. Transl. Vis. Sci. Technol..

[7] Förster A., Muqbel Z., Alkarkoukly S., Dick H.B., Taneri S. (2025). Nachbehandlung nach keratorefraktiver Lentikelextraktion (KLEx). Die Ophthalmol..

[8] Leccisotti A., Fields S.V., De Bartolo G. (2022). Refractive Corneal Lenticule Extraction on Previous Photorefractive Keratectomy, with Optical Coherence Tomography Study. Case Rep. Ophthalmol..

[9] Bteich Y., Assaf J.F., Gendy J.E., Awwad S.T. (2024). Keratorefractive Lenticule Extraction Using the Ziemer FEMTO LDV Z8 Platform (CLEAR): One-Year Results. J. Refract. Surg..

[10] Leccisotti A., Fields S.V., De Bartolo G. (2023). Femtosecond LASIK Retreatments After Primary Myopic Photorefractive Keratectomy. Cornea.

[11] Donate D., Thaëron R. (2015). Preliminary Evidence of Successful Enhancement After a Primary SMILE Procedure With the Sub-Cap-Lenticule-Extraction Technique. J. Refract. Surg..

[12] Sedky A.N., Wahba S.S., Roshdy M.M., Ayaad N.R. (2018). Cap-preserving SMILE Enhancement Surgery. BMC Ophthalmol..

[13] Kling S., Spiru B., Hafezi F., Sekundo W. (2017). Biomechanical Weakening of Different Re-treatment Options After Small Incision Lenticule Extraction (SMILE). J. Refract. Surg..

[14] Riau A.K., Liu Y.C., Lim C.H.L., Lwin N.C., Teo E.P., Yam G.H., Tan D.T., Mehta J.S. (2017). Retreatment strategies following Small Incision Lenticule Extraction (SMILE): In vivo tissue responses. PLoS ONE.

[15] Donate D., Thaëron R. (2019). SMILE With Low Energy Levels: Assessment of Early Visual and Optical Quality Recovery. J. Refract. Surg..

[16] Leccisotti A., Fields S.V., De Bartolo G. (2023). Refractive Corneal Lenticule Extraction With the CLEAR Femtosecond Laser Application. Cornea.

[17] Reinstein D.Z., Archer T.J., Carp G.I. (2018). The Surgeon’s Guide to SMILE: Small Incision Lenticule Extraction.

[18] Reinstein D.Z., Carp G.I., Archer T.J., Gobbe M. (2014). Outcomes of small incision lenticule extraction (SMILE) in low myopia. J. Refract. Surg..

[19] Reinstein D.Z., Carp G.I., Archer T.J., Vida R.S. (2018). Outcomes of Re-treatment by LASIK After SMILE. J. Refract. Surg..

[20] Alio Del Barrio J.L., Parafita-Fernandez A., Canto-Cerdan M., Alio J.L., Teus M. (2021). Evolution of corneal thickness and optical density after laser in situ keratomileusis versus small incision lenticule extraction for myopia correction. Br. J. Ophthalmol..

[21] Jain V., Mhatre K., Shome D. (2010). Flap buttonhole in thin-flap laser in situ keratomileusis: Case series and review. Cornea.

[22] Siedlecki J., Luft N., Kook D., Wertheimer C., Mayer W.J., Bechmann M., Wiltfang R., Priglinger S.G., Sekundo W., Dirisamer M. (2017). Enhancement After Myopic Small Incision Lenticule Extraction (SMILE) Using Surface Ablation. J. Refract. Surg..

[23] Kaiserman I., Sadi N., Mimouni M., Sela T., Munzer G., Levartovsky S. (2017). Corneal Breakthrough Haze After Photorefractive Keratectomy With Mitomycin C: Incidence and Risk Factors. Cornea.

[24] Wilson S.E. (2020). Biology of keratorefractive surgery- PRK, PTK, LASIK, SMILE, inlays and other refractive procedures. Exp. Eye Res..

[25] Liu Y., Shang J.M., Hu C., Chen X., Jiang W.S., Huang J. (2025). Early assessment of visual outcomes and corneal stability in eyes with a pre-planned residual stromal thickness of 280 to 300 µm following small incision lenticule extraction. Int. J. Ophthalmol..

[26] Primavera L., Canto-Cerdan M., Alio J.L., Alio Del Barrio J.L. (2022). Influence of age on small incision lenticule extraction outcomes. Br. J. Ophthalmol..

