# Supplementary material for: CLEAR Lenticule Extraction for Enhancement After Primary Lenticule Extraction Surgery
Source: J Clin Med. 2026 Jun 28;15(13):5036. doi: 10.3390/jcm15135036 (PMC13362835; doi:10.3390/jcm15135036)
Supplement: Supplementary file 1 [file jcm-15-05036-s001.zip › jcm-4382261-supplementary.pdf]

Supplementary Table S1. Parameters of the primary CLEAR treatment.

Patient	Eye Side	Mean Sim-K value (D)	Q value	Pachymetry at Thinnest Point (μm)	Optical Zone (mm)	Cap Thickness (μm)	Residual Stroma [1] (μm)
Patient 1	OD	43.20	-0.21	572	6.5	110	352
Patient 2	OD	43.00	-0.33	560	6.5	110	339
Patient 3	OD	41.90	-0.39	592	6.5	110	367
	OS	41.60	-0.26	583	6.5	110	359
Patient 4	OD	44.20	-0.38	615	6.5	120	368
Patient 5	OD	42.90	-0.39	593	6.5	110	329

[1] Residual stroma displayed on the FEMTO LDV Z8 monitor.

Supplementary Table S2. Tomography parameters measured preoperatively to primary CLEAR, preoperatively to secondary CLEAR, and postoperatively to secondary CLEAR.

Patient	Eye Side	Mean Sim-K (D)	Thinnest Pachymetry (μm)	CCT (μm)	Vertical Trefoil (μm)	Vertical Coma (μm)	Horizontal Coma (μm)	Oblique Trefoil (μm)	Spherical Aberration (μm)	HOA RMS (μm)
**Preoperative Primary CLEAR**										
Patient 1	OD	43.2	572	566	-0.080	-0.160	-0.109	-0.109	0.168	0.326
Patient 2	OD	43.0	560	564	0.032	-0.332	-0.105	-0.015	0.305	0.529
Patient 3	OD	41.9	592	562	0.193	-0.447	0.155	-0.060	0.254	0.596
Patient 3	OS	41.6	583	587	-0.104	0.102	-0.143	0.047	0.183	0.311
Patient 4	OD	44.2	615	599	-0.047	0.084	-0.061	0.009	0.111	0.435
Patient 5	OD	42.9	593	582	0.108	-0.107	-0.11	0.054	0.219	0.339
**Preoperative Secondary CLEAR**										
Patient 1	OD	39.0	488	473	0.106	-0.069	-0.141	0.073	-0.079	0.354
Patient 2	OD	38.2	500	470	0.144	-0.753	-0.030	0.029	-0.016	0.843
Patient 3	OD	36.1	489	479	0.074	-0.837	0.309	0.259	0.162	0.972
Patient 3	OS	35.4	484	471	0.126	-0.779	0.107	0.050	0.354	0.902
Patient 4	OD	38.5	517	497	-0.024	-0.226	-0.064	0.039	0.248	0.409
Patient 5	OD	36.6	469	459	0.208	-1.004	-0.040	-0.035	0.299	1.135
**1 Month Postoperative Secondary CLEAR**										
Patient 1	OD	N/A	430	431	0.124	0.216	-0.206	-0.041	-0.323	0.708
Patient 2	OD	N/A	411	411	0.082	-0.723	-0.161	0.073	-0.113	0.825
Patient 3	OD	N/A	420	420	1.107	-0.218	0.116	-0.560	0.196	1.502
Patient 3	OS	N/A	361	361	0.439	0.044	-0.011	-0.416	-0.252	0.932
Patient 4	OD	N/A	N/A	N/A	0.097	0.003	0.008	-0.039	0.061	0.398
Patient 5	OD	N/A	N/A	N/A	0.241	-0.719	0.136	-0.193	0.275	0.965
**3 Months Postoperative Secondary CLEAR**										
Patient 1	OD	N/A	437	440	0.095	0.098	-0.170	-0.094	-0.141	0.413
Patient 2	OD	N/A	424	428	N/A	N/A	N/A	N/A	N/A	N/A
Patient 3	OD	N/A	N/A	N/A	-0.010	-0.661	0.104	-0.109	0.089	0.810
Patient 3	OS	N/A	N/A	N/A	0.268	-0.142	0.421	-0.092	0.029	0.717
Patient 4	OD	N/A	N/A	N/A	0.027	-0.167	0.018	0.000	0.113	0.502
Patient 5	OD	N/A	437	440	0.158	-0.574	0.001	0.058	0.344	0.771

CCT = central corneal thickness; CLEAR = Corneal Lenticule Extraction for Advanced Refractive Correction; HOA RMS = higher-order aberration root mean square
